# A Generalizable Multimodal Model for Treatment‐Stratified Risk and Survival Assessment under Real‐World Constraints: A Multi‐Center Study of Colorectal Cancer

**DOI:** 10.1002/advs.78019

**Published:** 2026-09-29

**Authors:** Chuangjie Cao, Zheyi Ji, Chang He, Jiying Wang, Xiaoming Luo, Junyi Liu, Xiaohu Jing, Fang Yan, Yirong Chen, Linhao Qu, Sen Yang, Le Lu, Yuming Jiang, Xiyue Wang, Chengyun Dou, Junhan Zhao

**Affiliations:** ^1^ Department of Pathology, the First Affiliated Hospital University of South China Hengyang Hunan China; ^2^ Comprehensive Cancer Center UChicago Medicine Chicago USA; ^3^ Department of Human Genetics The University of Chicago Chicago USA; ^4^ Committee on Genetics, Genomics and Systems Biology The University of Chicago Chicago USA; ^5^ Changsha Taifang Yaoyu Technology Co., Ltd. Changsha Hunan China; ^6^ Hengyang Medical School University of South China Hengyang Hunan China; ^7^ Changsha Chuangke Software Co., Ltd. Changsha Hunan China; ^8^ Shanghai AI Laboratory Shanghai China; ^9^ Department of Biomedical Informatics Harvard Medical School Boston USA; ^10^ Ant Healthcare (AFU), Ant Group Hangzhou Zhejiang China; ^11^ Department of Radiation Oncology Wake Forest University School of Medicine Winston‐Salem USA; ^12^ Department of Infectious Diseases The First Affiliated Hospital University of South China Hengyang Hunan China; ^13^ Department of Pediatrics The University of Chicago Chicago USA

**Keywords:** bioinformatics, computational biology, gastroenterology, machine learning, medicine, pathology

## Abstract

Integrating histopathology, genomic, and clinical phenotypes holds promise for improving prognostic patient stratification, including within treatment‐defined subgroups, in colorectal cancer (CRC). However, ensuring the availability of all modalities in routine clinical practice remains challenging. Comprehensive molecular profiling is often constrained by cost and turnaround time, leaving histopathology as the most consistently available modality across institutions. In addition, variability in staining protocols, scanning devices, patient demographics, and outcome distributions across centers undermines the generalizability of models trained under controlled conditions with complete inputs. To address these challenges, we developed the Domain‐Adaptive Incomplete Multimodal Stratification framework (DAIMS). DAIMS leverages paired histopathology, genomic, and clinical data during training to learn generalizable disease representations and uses histology‐derived surrogate latent representations when auxiliary modalities are unavailable, enabling flexible histology‐only inference. Trained on TCGA samples and validated on two independent European and East Asian cohorts comprising 842 patients, DAIMS consistently outperformed state‐of‐the‐art methods. Adapted DAIMS improved the C‐index by 5.6%–13.9% on F1CRC and 4.1%–9.0% on SURGEN compared with baseline methods. DAIMS improved within‐cohort prognostic discrimination and localized prognostically relevant morphological patterns that remained stable across institutions. Our evaluation showed that DAIMS achieved generalizable survival stratification across disease stages, molecular subgroups, and treatment‐defined patient groups.

## Graphical abstract

DAIMS is a multimodal model for treatment stratified risk and survival assessment in colorectal cancer. Training draws on pathology, genomics, and clinical reports, yet inference requires only whole slide images, and adaptation to new cohorts proceeds without target survival outcomes. Across multiple centers, stratification remains accurate when modalities are missing, bringing multimodal prognostication within reach of routine practice.

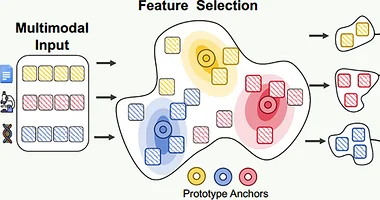

**Abbreviations**
CRC
: Colorectal cancer
DAIMS
: Domain‐Adaptive Incomplete Multimodal Stratification
WSI
: Whole‐slide image
TCGA
: The Cancer Genome Atlas
LLM
: Large language model
KM
: Kaplan‐Meier
C−index
: Concordance index
OA
: Overlap area
MSI
: Microsatellite instability
TMB
: Tumor mutational burden
MMR
: Mismatch repair
H&E
: Hematoxylin and eosin
MHA
: Multi‐head attention
AR
: Anterior resection
APR
: Abdominoperineal resection
BRAF
: B‐Raf proto‐oncogene, serine/threonine kinase
KRAS
: Kirsten rat sarcoma viral oncogene homolog

## 1 Introduction

Colorectal cancer (CRC) remains a major global health burden, accounting for approximately 1.93 million new cases and nearly 935,000 deaths each year [1, 2, 3]. Despite continued advances in staging systems and therapeutic strategies, survival outcomes remain highly heterogeneous even among patients with similar clinicopathological profiles [2, 4, 5]. This heterogeneity reflects the multifactorial nature of CRC progression, often shaped by the interplay of tumor stage, molecular alterations, and histopathological phenotypes [6, 7, 8, 9]. Improving prognostic modeling therefore requires integrative approaches that can capture complementary information across biological and clinical levels.

In routine clinical practice, histopathological slides remain central to the diagnosis and assessment of CRC. Histology images encode critical morphological features [3, 10, 11, 12]. In contrast, sequencing‐based genomic profiles capture driver mutations (e.g., *KRAS* [13] and *BRAF* [14]), microsatellite instability (MSI), tumor mutational burden (TMB), and copy number alterations, molecular features closely associated with disease progression and survival [15, 16, 17]. Clinical records add a longitudinal dimension, capturing family history, treatment course and response, and laboratory measurements that further inform individual risk assessment [18, 19]. Together, these complementary clinical data modalities offer a rich profile of patient disease status.

Recent advances in prognostic modeling have been achieved across individual data modalities, including histopathological image analysis [20, 21], genomic profiling [22, 23, 24], and clinical text [25, 26, 27] modeling. Several pathology foundation models have advanced transferable histopathological representation learning through large‐scale pretraining and knowledge distillation, such as MUSK [28], H‐Optimus‐1 [29], and GPFM [30]. Motivated by the complementary nature of these heterogeneous data sources, multimodal deep learning frameworks have been proposed to integrate histopathological images, genomic features, and clinical information [31, 32, 33, 34]. However, most existing approaches assume that the training and deployment environments share the same modality set, acquisition pipeline, cohort composition, and outcome distribution [35, 36]. This assumption rarely holds in practice, where two interrelated challenges limit clinical translation.

In real‐world clinical environments, multimodal inputs are often incomplete and heterogeneous, which limits the reliability of trained models at deployment [37]. First, visual covariate shift arises from staining protocols, scanners, microns‐per‐pixel resolution, image quality, and tissue‐processing differences across institutions [38, 39, 40]. Second, cohort and label‐space shift arise because patient populations, treatment pathways, censoring patterns, survival endpoints, and event‐time distributions can differ between source and external cohorts. These differences are especially important for survival models, because a risk head optimized on source‐domain event‐time bins may be poorly calibrated when the target cohort follows a different endpoint or time distribution. Third, modality availability itself varies across domains: many hospital datasets contain only histopathological slides and limited clinical metadata, whereas genomic profiles may be unavailable because of cost and long processing times [41, 42, 43]. Clinical reports are also frequently stored as unstructured narratives, complicating standardization and large‐scale computational analysis [18, 44]. Consequently, morphological, molecular, and clinical information are often fragmented and only partially observed rather than forming a unified patient representation. Modality incompleteness and multi‐source batch effects therefore jointly limit the robustness and clinical applicability of existing multimodal prognostic models, underscoring the need for frameworks that remain reliable under incomplete and heterogeneous real‐world conditions [11].

Here, we propose the Domain‐Adaptive Incomplete Multimodal Stratification framework (DAIMS). DAIMS is designed as a domain adaptation framework for real‐world CRC patient stratification. It uses multimodal TCGA data to learn transferable representations, then adapts a WSI‐centered deployment branch to external cohorts where only pathology slides are available. Within this design, prototype‐guided aggregation and fusion, cross‐modal representation imputation, and lightweight target‐domain fine‐tuning are not independent goals. They are complementary mechanisms for aligning patient representations across heterogeneous visual, modality‐availability, and outcome‐distribution shifts [45, 46]. DAIMS is trained on 351 TCGA samples and evaluated on two external cohorts (F1CRC, n=420; SURGEN [47], n=422), consistently outperforming both multimodal and unimodal baselines across multiple encoders.

## 2 Results

### 2.1 Overview of the Domain‐Adaptive Incomplete Multimodal Stratification (DAIMS) Framework

We present DAIMS (Figure 1), a multimodal survival framework for colorectal cancer. In clinical practice, comprehensive molecular profiling is often delayed or unavailable. To address this, DAIMS is designed to operate on histology alone at inference, while leveraging multimodal training (histology, molecular profiles, and clinical notes) to enrich the model's learned representations. We formulate the resulting modality‐availability shift between training and inference as a domain adaptation problem [37, 38, 39]. DAIMS employs an imputation module to encourage WSI features to incorporate genomic and clinical information during training [41, 42] (Figure 1b–d). At transfer time, a light‐weight target‐domain adaptation step is performed where only the WSI adapter and survival head are updated based on pseudo risk labels obtained from target domain WSIs by the frozen source‐domain DAIMS model. Crucially, this adaptation step does not use the target‐domain survival time, censoring status, event labels, or risk‐group annotations. Target‐domain survival outcomes were reserved exclusively for final evaluation. We assessed DAIMS in terms of prognostic discrimination, transferability to external cohorts, treatment‐stratified analysis, and molecular‐subgroup stratification.

**FIGURE 1:**
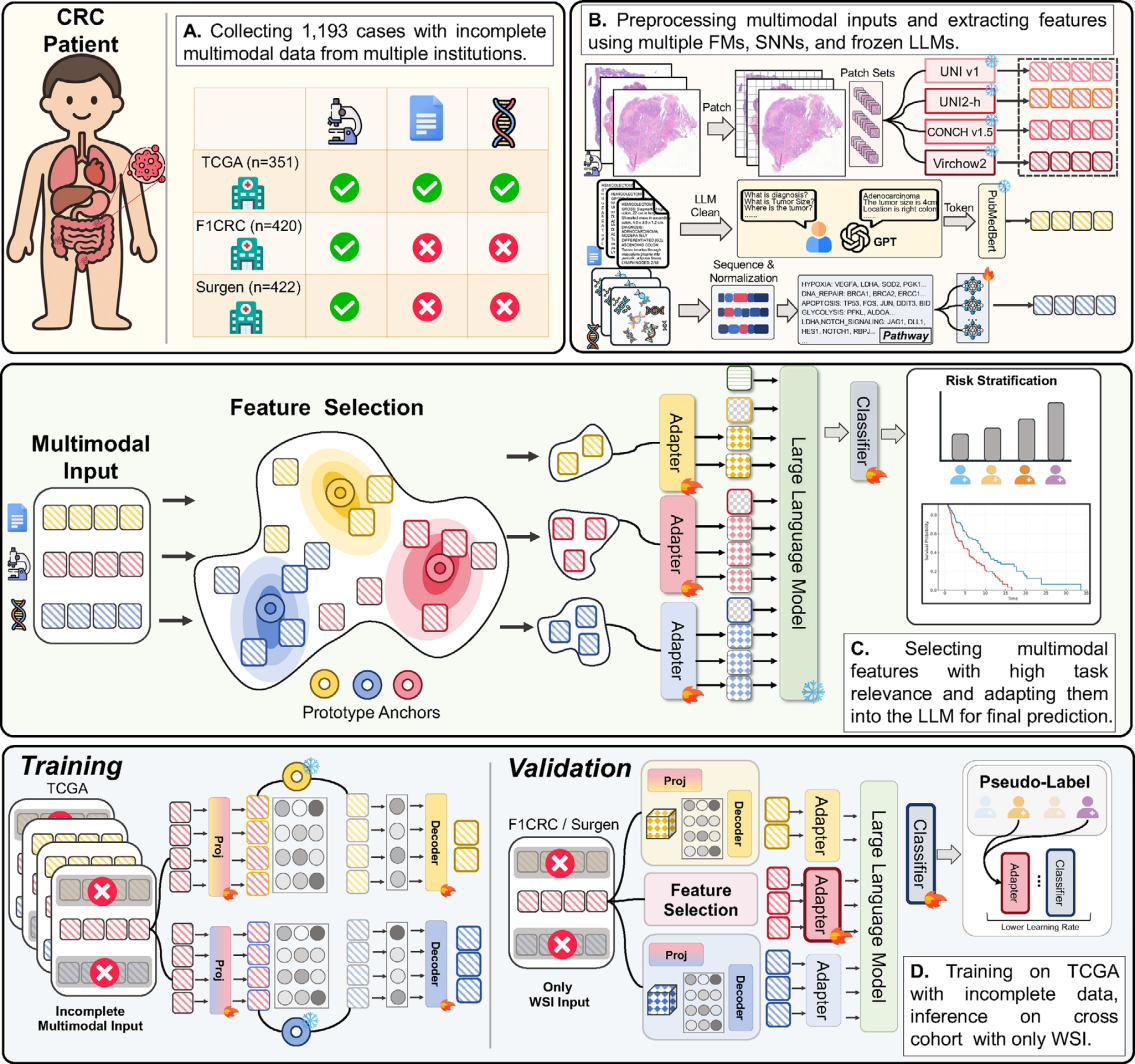
Overview of DAIMS. (A) The TCGA cohort (n=351) contains whole‐slide images (WSIs), genomics, and clinical reports, whereas the external F1CRC (n=420) and SURGEN (n=422) cohorts provide WSIs for model inference, with selected clinical or molecular annotations used for subgroup evaluation. (B) Multimodal data preprocessing. WSIs are tiled and encoded using pathology foundation models. Clinical reports are converted into structured text representations and embedded with biomedical language models, while genomic data are summarized as pathway‐level features. (C) DAIMS architecture. A feature selection module extracts informative representations from each modality and maps them into a shared latent token space processed by a frozen large language model (LLM) to produce patient‐level prognostic risk predictions. (D) Training and deployment strategy. During multimodal training on TCGA, representation imputation modules encourage WSI features to capture complementary genomic and clinical information. When transferring to external cohorts with pathology‐only data, a lightweight domain adaptation step uses model‐generated pseudo risk labels to update the WSI adapter and prediction head, improving cross‐cohort prognostic risk ordering without requiring target‐domain genomic or clinical‐report inputs.

### 2.2 Multi‐Center and Multi‐Modal Dataset and Cohort Characteristics

We collected and analyzed three independent colorectal cancer (CRC) cohorts: TCGA (n=351), F1CRC (n=420), and SURGEN (n=422) (Table 1). TCGA served as the source‐domain cohort and provided matched WSIs, genomic pathway profiles, and structured clinical reports. In contrast, F1CRC and SURGEN provided WSIs as model inputs for external validation, reflecting real‐world modality‐availability shift, while additional clinical or molecular annotations were used only for subgroup analyses [48, 49]. For downstream subgroup analysis, TCGA and F1CRC additionally contained detailed treatment annotations, including radiation therapy, pharmaceutical treatment, chemotherapy regimens, and surgical procedures. SURGEN further provided molecular biomarker information, including *BRAF* [50], *KRAS* exon 2 [51], and mismatch repair (MMR) status [52], together with complete pathological staging information. The three cohorts exhibited substantial demographic, clinical, and technical heterogeneity. Molecular annotation also varied across cohorts: TCGA provided comprehensive molecular profiling, whereas F1CRC did not provide equivalent molecular profiling and SURGEN included selected biomarkers only. TCGA comprised a racially diverse population and was predominantly composed of White patients (71.2%), whereas F1CRC consisted entirely of East Asian patients. In contrast, SURGEN was derived from a United Kingdom cohort predominantly representing Caucasian patients. Clinical characteristics also differed substantially across cohorts. For example, rectal tumors accounted for 48.6% of cases in F1CRC but 10.0% in TCGA, while SURGEN contained a larger proportion of Stage III disease (45.5%) than TCGA (29.6%). In addition, differences in slide preparation, staining protocols, and scanner platforms across institutions introduced substantial inter‐center variation in histopathology appearance [53]. Together, these complementary cohorts provide a realistic benchmark for evaluating multimodal survival models under heterogeneous cross‐cohort settings.

**TABLE 1:** Patient characteristics of study cohorts.

Patient characteristics	TCGA	F1CRC	SURGEN^a^
**Number of patients**	N=351	N=420	N=422
**Age at diagnosis**	64.5±13.0	62.9±10.9	68.0±12.0
**Sex**			
Male	184 (52.4%)	252 (60.0%)	227 (53.8%)
Female	167 (47.6%)	168 (40.0%)	195 (46.2%)
**Race**			
Black or African American	59 (16.8%)	0 (0.0%)	—
White	250 (71.2%)	0 (0.0%)	—
Asian	12 (3.4%)	420 (100.0%)	—
Native American or Alaska Native	1 (0.3%)	0 (0.0%)	—
Pacific Islander	0 (0.0%)	0 (0.0%)	—
U/A	29 (8.3%)	0 (0.0%)	—
**Tumor Location** ^b^			
Proximal Colon	171 (48.7%)	81 (19.3%)	139 (32.9%)
Distal Colon	142 (40.5%)	79 (18.8%)	157 (37.2%)
Rectum	35 (10.0%)	204 (48.6%)	123 (29.1%)
U/A	3 (0.9%)	56 (13.3%)	3 (0.7%)
**Stage**			
Stage I	55 (15.7%)	70 (16.7%)	59 (14.0%)
Stage II	129 (36.8%)	73 (17.4%)	151 (35.8%)
Stage III	104 (29.6%)	122 (29.0%)	192 (45.5%)
Stage IV	48 (13.7%)	12 (2.9%)	20 (4.7%)
U/A	15 (4.3%)	143 (34.0%)	0 (0.0%)

^a^ A detailed racial breakdown for the SURGEN cohort is not available, but the source indicates that it is majority Caucasian.

^b^ Tumor location was summarized at the patient level. Proximal colon includes cecum/caecum, ascending colon, hepatic flexure, and transverse colon. Distal colon includes splenic flexure, descending colon, sigmoid colon, and rectosigmoid junction. Rectum includes rectal tumors. U/A denotes missing, not reported, or nonspecific colon‐only annotations.

### 2.3 DAIMS Achieves Robust Risk Stratification Across Diverse Cohorts

The prognostic performance of DAIMS was first evaluated on the TCGA source cohort under both fully observed and controlled modality‐availability conditions. The framework was then assessed for cross‐domain generalization on the F1CRC and SURGEN cohorts, where only WSI data were used for model inference.

#### 2.3.1 Source‐Domain Evaluation Under Modality‐Availability Shifts

DAIMS was trained with four pathology foundation encoders to assess whether the observed performance gains were preserved across heterogeneous WSI backbones. Under fully observed multimodal inputs, DAIMS consistently separated predicted high‐risk and low‐risk patients in Kaplan–Meier analysis (Figure 2A). CONCH v1.5 achieved the strongest stratification (log‐rank $p=1.20\times 10^{-3}$), while UNI v1, UNI2‐h, and Virchow2 showed the same overall trend. This pattern was reproduced across multiple pathology encoders, arguing against the gain being restricted to a single backbone configuration [7, 54, 55, 56].

**FIGURE 2:**
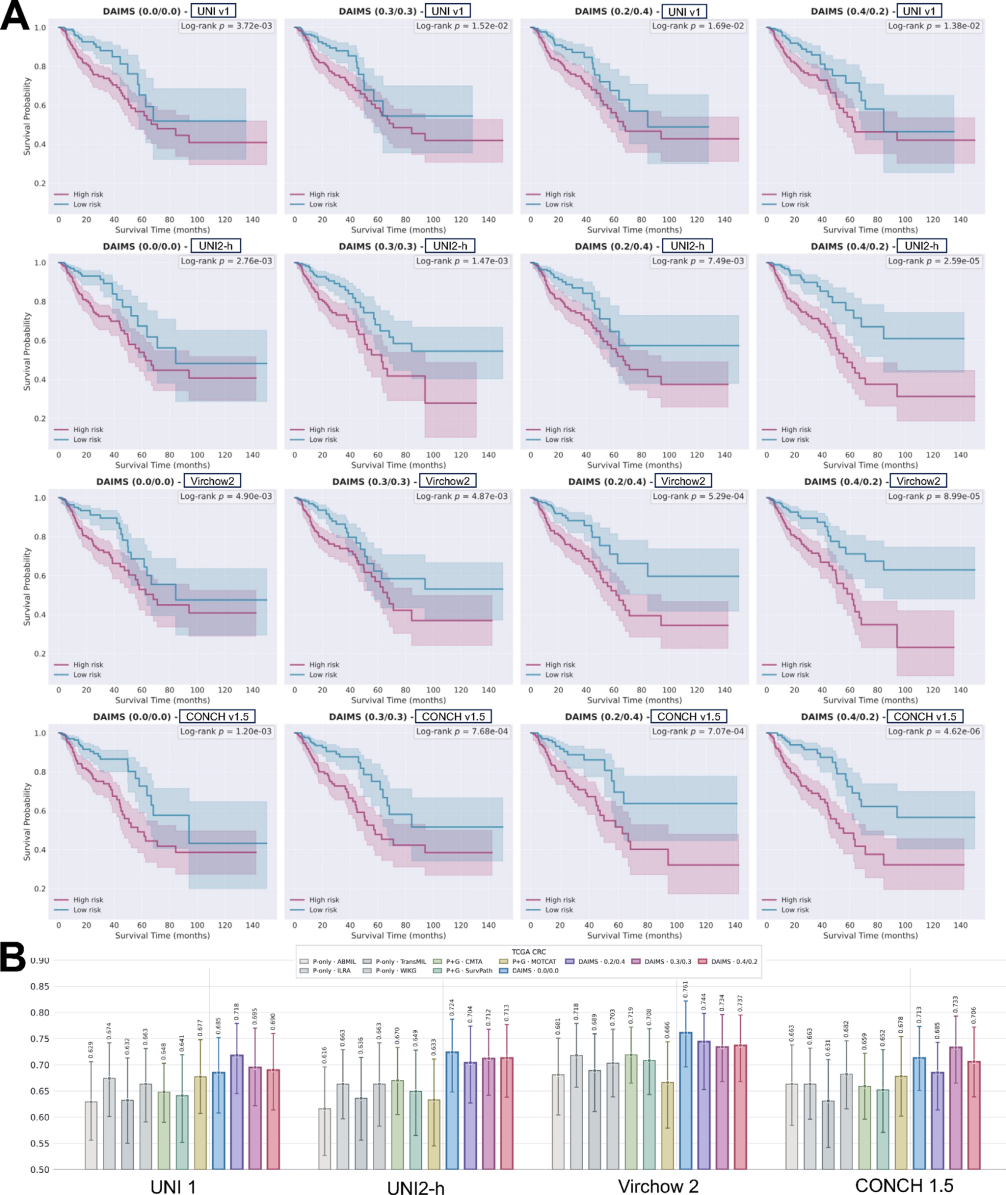
Evaluation of DAIMS under controlled modality‐availability shifts and comparison with existing prognostic models. (A) Kaplan–Meier analysis on the TCGA colorectal cancer cohort showing the robustness of DAIMS across varying modality‐availability ratios. The panels correspond to (from left to right): full modality, (30%, 30%), (20%, 40%), and (40%, 20%) (genomic, report). Across all settings, DAIMS consistently separates high‐ and low‐risk groups, demonstrating stable patient stratification under modality‐availability shifts. (B) Comparative performance of DAIMS against seven prior approaches, including four pathology‐only methods and three pathology–genomics models. Shown are C‐index evaluated on the TCGA cohort. DAIMS achieves competitive or superior prognostic accuracy across both single‐modality and multimodal baselines, underscoring its capacity to effectively integrate heterogeneous source‐domain information and maintain performance under modality‐availability shift. Bootstrap 95% confidence intervals for the primary DAIMS C‐index estimates are provided in Figure S1 and Table S5.

DAIMS was further evaluated under three controlled modality‐availability conditions, in which genomic and clinical reports data were removed at missing ratios of (30%, 30%), (20%, 40%), and (40%, 20%) for (genomic, clinical reports), respectively, while always retaining WSI data. Complete fold‐wise results are provided in Table S1. Across all four encoders and all missing‐ratio settings, Kaplan–Meier analysis continued to demonstrate significant survival stratification. Using CONCH v1.5 features, the (30%, 30%) setting yielded $p=7.68\times 10^{-4}$, the (20%, 40%) setting yielded $p=7.07\times 10^{-4}$, and the (40%, 20%) setting yielded $p=4.62\times 10^{-6}$. In general, performance degradation relative to the fully observed setting was limited across these auxiliary‐modality imbalance settings, indicating that DAIMS can retain informative prognostic signals even when auxiliary modalities are partially unavailable. Notably, the relative contribution of genomic and report availability varied across encoders and missing‐ratio settings, but the overall results suggest that auxiliary genomic and clinical‐report supervision provides complementary information for risk stratification [41, 42].

Quantitative comparative experiments further supported these observations (Figure 2B; Table S2). DAIMS outperformed pathology‐only baselines across all evaluated settings while maintaining stable performance under incomplete modality conditions. Using CONCH v1.5 features, the mean C‐index reached 0.761 under fully observed inputs, exceeding ABMIL (0.681), ILRA (0.718), and WIKG (0.703), and remained at 0.744 under the (20%, 40%) missing‐ratio setting. Similar trends were observed with UNI v1, UNI2‐h, and Virchow2, whose pathology‐only baseline performance ranged from 0.62 to 0.68 but increased to 0.685–0.733 after integration into DAIMS. Overall, DAIMS maintained prognostic discrimination across heterogeneous pathology encoders and under reduced auxiliary‐modality availability.

To directly assess inference‐time patch sensitivity, we evaluated fixed DAIMS models from all four pathology foundation encoders. Patient‐level prognostic discrimination was reduced at low patch budgets but broadly maintained from 5,000 to 10,000 patches per WSI, without a consistent gain beyond the default 4,096‐patch setting (Figure S5 and Table S7). A complementary analysis varying the maximum patch budget during model development and held‐out evaluation showed the same non‐monotonic pattern across source missingness settings.

#### 2.3.2 Cross‐Domain Generalization to Independent Cohorts

The ability of DAIMS to generalize from TCGA to independent external cohorts was evaluated on F1CRC (n=420) and SURGEN (n=422). These cohorts differ from TCGA in patient demographics, specimen preparation, staining protocols, scanning hardware, image quality, and survival‐time distributions [53], providing a stringent setting for cross‐domain and cross‐population evaluation.

Because survival endpoints and event‐time distributions differed across cohorts, high‐ and low‐risk groups were defined separately within each cohort using the cohort‐specific median predicted risk score. Across both F1CRC (Figure 3A) and SURGEN (Figure 3C), Kaplan–Meier analysis showed significant separation between predicted high‐risk and low‐risk groups under all encoder configurations and all missing‐ratio settings (all log‐rank p<0.05). In SURGEN, the strongest separation was observed with UNI2‐h under the (20%, 40%) setting ($p=3.98\times 10^{-17}$), while Virchow2 achieved strong stratification under the (40%, 20%) setting ($p=2.20\times 10^{-15}$). In F1CRC, CONCH v1.5 under the (20%, 40%) setting yielded the most significant separation ($p=3.62\times 10^{-9}$), and UNI v1 also achieved strong stratification under the (20%, 40%) setting ($p=6.53\times 10^{-8}$). No single encoder or missing‐ratio configuration clearly outperformed across both cohorts, indicating stable prognostic stratification across heterogeneous deployment conditions.

**FIGURE 3:**
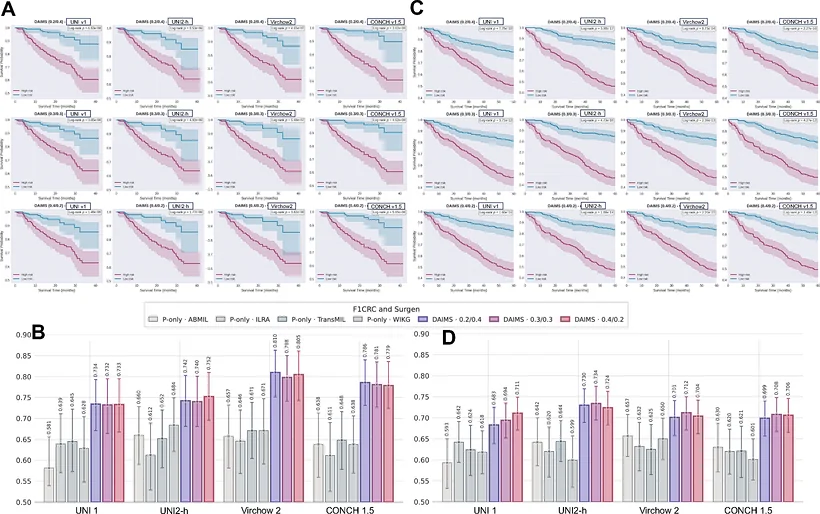
Cross‐domain validation of DAIMS models trained under different modality‐availability scenarios and evaluated on external datasets. (A,C) Kaplan–Meier survival curves for the F1CRC and SURGEN cohorts using DAIMS models trained under three controlled modality‐availability conditions. Each row corresponds to a missing‐rate configuration, and each column shows results for a different pathology encoder. High‐ and low‐risk groups were defined separately within each external cohort using the cohort‐specific median predicted risk score. Across all encoders and settings, DAIMS preserved separation between high‐ and low‐risk groups, demonstrating superior generalization and cross‐domain adaptation despite pathology‐only target‐domain inference. (B,D) Comparative C‐index performance on the F1CRC and SURGEN cohorts against four pathology‐only baselines. DAIMS outperforms all baselines, highlighting the advantage of multimodal source‐domain learning and lightweight adaptation under modality‐availability shift. Bootstrap 95% confidence intervals for the primary DAIMS C‐index estimates are provided in Table S5.

C‐index analysis was further performed to assess prognostic performance across external cohorts (Figure 3B,D; Table S2). After pseudo‐label‐based adaptation on each target cohort, DAIMS generally outperformed the corresponding pathology‐only baselines across encoders. In F1CRC, the best performance reached 0.810 with CONCH v1.5, followed by Virchow2 (0.786), UNI2‐h (0.752), and UNI v1 (0.734). In SURGEN, DAIMS achieved up to 0.734 (UNI2‐h), 0.712 (CONCH v1.5), 0.708 (Virchow2), and 0.711 (UNI v1), exceeding the corresponding pathology‐only baselines within each encoder group. The relative ranking of encoders was slightly different between cohorts, but DAIMS retained gains over pathology‐only models in all settings. Importantly, these results were obtained without access to target‐domain genomic or structured clinical reports data during inference. This suggests that multimodal training may enhance the robustness of pathology‐based representations under external deployment settings, particularly when only WSI data are available.

### 2.4 DAIMS Preserves Prognostic Stratification Across Treatment‐Defined and Clinical‐Molecular Subgroups

#### 2.4.1 Treatment Subgroups in TCGA

Patients were grouped by treatment modality (Radiation, Pharmaceutical therapy, Chemotherapy, and Surgery) and stratified into predicted high‐ and low‐risk subgroups within each category (Figure 4, top; Table S8). Kaplan–Meier analysis showed statistically significant survival separation in the Radiation, Pharmaceutical therapy, Chemotherapy, and Surgery groups. DAIMS captured residual prognostic risk within patient groups defined by broad treatment categories. Patients undergoing the same treatment modality can differ significantly in tumor differentiation, disease burden, microenvironment composition, and treatment sensitivity, all of which may leave morphological imprints on routine pathology slides [5, 57, 58].

**FIGURE 4:**
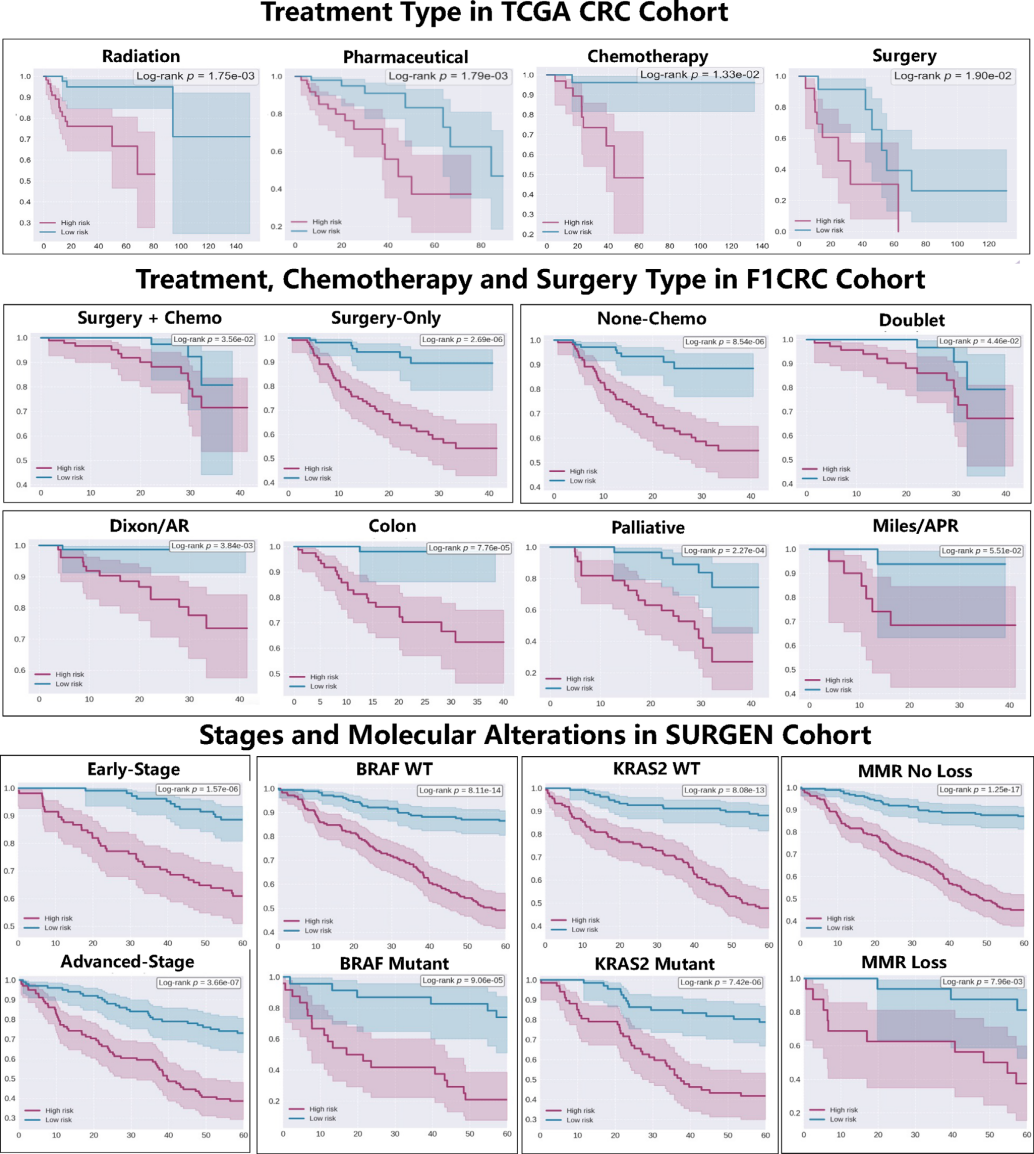
Kaplan–Meier survival stratification across different subgroups. From top to bottom, panels show results from the TCGA, F1CRC, and SURGEN cohorts. Within each prespecified subgroup, patients were dichotomized at the median DAIMS‐predicted risk score calculated within that subgroup. In TCGA, patients were stratified by treatment type and divided into DAIMS‐predicted high‐ and low‐risk groups. In F1CRC, DAIMS achieved significant survival separation across treatment modality, chemotherapy, and surgical subgroups. In SURGEN, significant stratification was observed across disease stage and genomic subgroups, including BRAF, KRAS exon 2, and MMR status (log‐rank test, all p<0.05).

#### 2.4.2 Treatment and Surgical Subgroups in F1CRC

Patients were grouped by treatment strategy into Surgery + Chemotherapy vs. Surgery‐only cohorts (Figure 4, middle; Tables S9–S11). In both groups, DAIMS produced clear and statistically significant separation between predicted high‐ and low‐risk patients, supporting transfer of the TCGA‐trained risk signal to an East Asian external cohort. Patients were further stratified by chemotherapy regimen into those who did not receive chemotherapy and those treated with doublet regimens (e.g., XELOX, CAPEOX, FOLFOX) [59]. DAIMS successfully distinguished survival outcomes within both subgroups.

Surgical stratification was further refined into four clinically meaningful categories: rectal resection with sphincter preservation (Dixon/AR), colon resection, palliative procedures (ostomy, exploratory surgery, bypass, stenting), and rectal resection without sphincter preservation (Miles/APR) [58]. DAIMS consistently stratified patients into high‐ and low‐risk groups with statistically significant survival differences across all four surgical subgroups, suggesting that morphology‐linked prognostic signals remain informative across clinically heterogeneous surgical subgroups.

#### 2.4.3 Stage and Molecular Subgroups in SURGEN

DAIMS achieved statistically significant separation between predicted high‐ and low‐risk patients within both early‐stage (I–II) and advanced‐stage (III–IV) disease in the SURGEN cohort (Figure 4, bottom; Tables S12–S15), indicating that prognostic discrimination was preserved even after controlling for this dominant clinical determinant [2, 60]. Beyond disease stage, we examined three molecular markers that define clinically distinct CRC subtypes. For BRAF mutation status, a defining alteration of a biologically aggressive CRC subtype [50], patients were stratified as WT vs. Mutant; DAIMS separated high‐ vs. low‐risk patients within both subgroups, including BRAF‐mutant tumors. For KRAS exon 2 mutation status [51, 61], patients were stratified as WT vs. Mutant, and DAIMS separated high‐ and low‐risk patients within each subgroup. For mismatch repair (MMR) status [52], patients were categorized as MMR‐proficient (pMMR) vs. MMR‐deficient (dMMR), and DAIMS separated high‐ vs. low‐risk patients in both subgroups [61].

**FIGURE 5:**
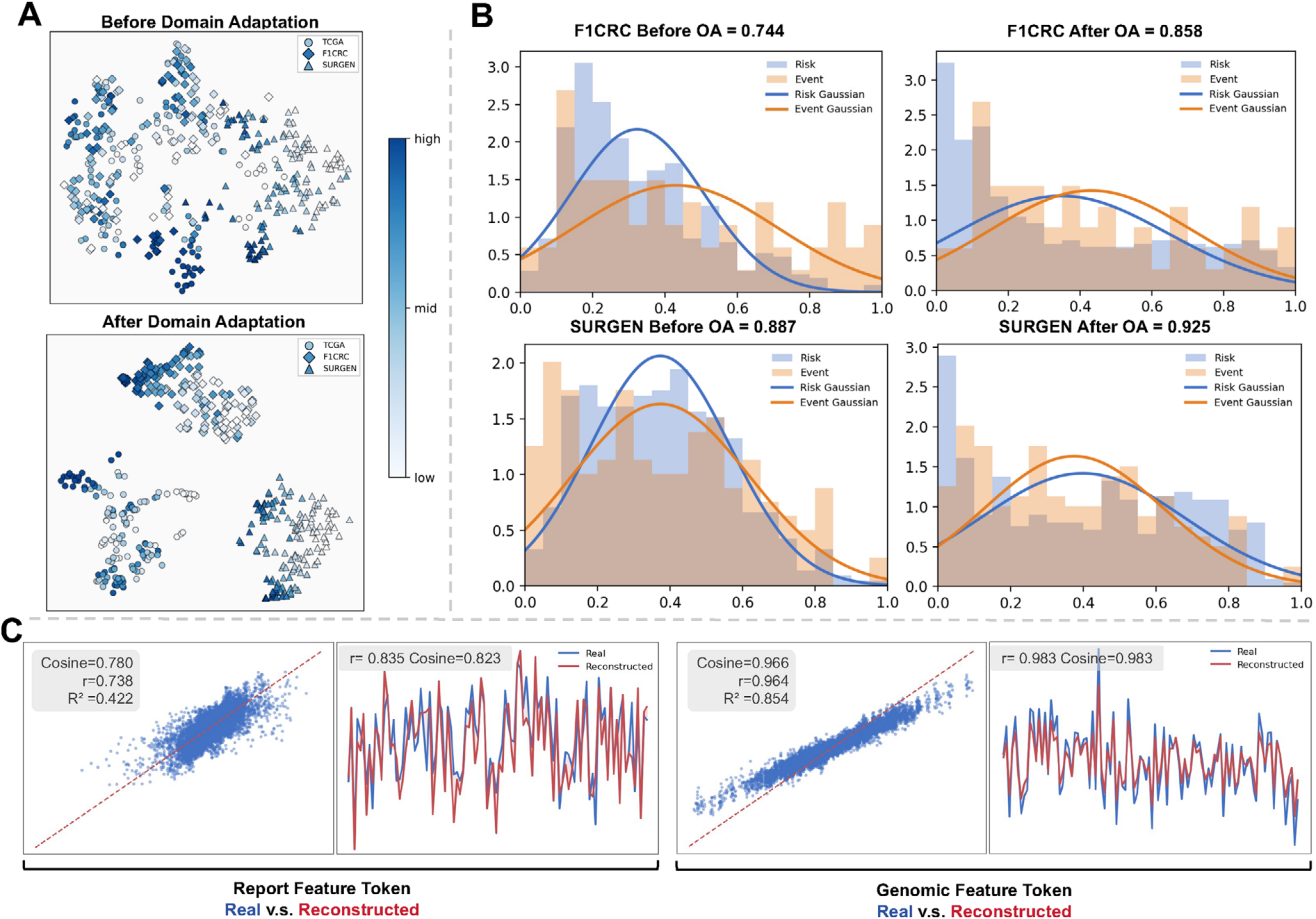
Domain adaptation and modality representation imputation. (A) t‐SNE [62] visualization of WSI‐derived features from TCGA, F1CRC, and SURGEN before and after domain adaptation. Pre‐adaptation features showed cohort separation and weak risk structure. After adaptation, cohort identity remained visible, but clearer within‐cohort risk gradients emerged, indicating improved prognostic organization without complete cross‐cohort feature alignment. (B) Gaussian‐fitted distributions of predicted risks and observed event times show increased distributional overlap after adaptation, with OA increasing from 0.744 to 0.858 in F1CRC and from 0.887 to 0.925 in SURGEN. OA is reported as an exploratory distributional‐alignment measure. (C) Representation imputation performance differs across modalities. Genomic pathway features are reconstructed with high fidelity (cosine 0.966, r=0.964, $R^{2}=0.854$), whereas clinical‐reports embeddings show moderate agreement (cosine 0.780, r=0.738, $R^{2}=0.422$), reflecting the structured nature of pathway‐level genomic signals compared with the greater variability of short language‐derived clinical embeddings.

Together, these analyses test whether the prognostic signal captured by DAIMS primarily recapitulates known clinical and molecular stratifiers or instead provides additional prognostic information that remains stable across clinically defined subgroups. The stable stratification across subgroups defined by BRAF, KRAS, MMR, stage, and treatment argues against the learned risk signal being solely explained by the examined clinical or molecular factors. This indicates that histopathologic patterns provide prognostic information not fully captured by canonical clinical classifications, which is reflected in the representations learned by DAIMS through multimodal source‐domain training. Exact sample sizes for all treatment‐, stage‐, and molecularly defined subgroups are provided in Tables S12–S15.

### 2.5 Domain Adaptation Improves WSI‐Derived Risk Organization Across External Cohorts

To examine the effect of lightweight adaptation on external deployment, we analyzed the organization of WSI‐derived semantic representations across TCGA, F1CRC, and SURGEN cohorts before and after adaptation. During WSI‐only inference, DAIMS generates histology‐derived surrogate tokens for multimodal fusion. The lower clinical‐report $R^{2}$ reflects the heterogeneity of free‐text embeddings while these tokens retain task‐relevant information for survival prediction. These cohorts differ in population composition, batch effects from fixation, staining, and scanning, and the survival prediction head optimized on TCGA‐specific event‐time bins, leading to distribution mismatch in external cohorts [63, 64].

Before adaptation, t‐SNE projections (Figure 5A) showed that TCGA and F1CRC samples were largely mixed, without an obvious low‐to‐high predicted‐risk organization, whereas SURGEN samples formed a distinct cluster shifted relative to the other cohorts. Within each cohort, high‐risk and low‐risk cases were poorly separated. In F1CRC, risk predictions were generally underestimated, whereas SURGEN predictions were concentrated around the median, indicating limited transferability of the TCGA‐trained survival mapping under unadjusted conditions.

After adaptation, cohort‐specific structure remained visible, while risk gradients became clearer within each cohort. This pattern is consistent with task‐oriented adaptation that improves prognostic organization while preserving meaningful cross‐cohort differences. As t‐SNE provides a qualitative visualization, generalizability was evaluated by prognostic discrimination across independent cohorts. We further quantified this improvement by comparing Gaussian‐fitted predicted‐risk distributions with observed event‐time distributions (Figure 5B). Their agreement was measured using the overlap area (OA), defined as OA=∫min(p(x),q(x))dx, where p(x) and q(x) denote normalized predicted‐risk and event‐time densities. The OA increased from 0.744 to 0.858 in F1CRC and from 0.887 to 0.925 in SURGEN. The increase in OA was larger in F1CRC (0.114) than in SURGEN (0.038). OA characterizes the distributional alignment of relative prognostic risk. The observed OA increases indicate more coherent within‐cohort risk organization after adaptation. Complementing this analysis, IPCW Brier scores decreased after adaptation at all six evaluated horizons in both F1CRC and SURGEN, indicating lower time‐dependent survival‐probability prediction error (Table S6). Together, these analyses support improved risk organization and outcome‐based survival prediction after adaptation.

We evaluated pseudo‐label quality through its downstream effect on model performance, averaging over all four pathology encoders and all missing modality scenarios at each of the eight adaptation iterations. In F1CRC, the mean C‐index gain increased steadily across iterations and reached 0.076 at iteration 8. In SURGEN, the mean gain increased over the first five iterations and then plateaued, ending at 0.039 at iteration 8. Median split risk group separation increased in both cohorts (Figure S2). Since pseudo‐labels are regenerated at every iteration, compounding pseudo‐label error would produce a decline in later iterations, which we do not observe in either cohort. A focused sensitivity analysis evaluated adaptation iterations, learning rate, update scope, and the target‐domain case selection ratio. Learning‐rate changes produced modest effects. Joint prediction‐head/WSI‐adapter updating and the default selection ratio of 0.5 provided balanced performance across cohorts. Eight adaptation iterations improved within‐cohort discrimination relative to four iterations in both cohorts (Figure S3). Together, these results indicate that lightweight adaptation improves within‐cohort risk organization and supports prognostic discrimination across heterogeneous multicenter CRC cohorts. DAIMS provides cohort‐relative prognostic rankings and adapts to a new institution using only target‐domain WSIs and model‐generated pseudo‐risk labels [45, 46, 65, 66]. Labeled target‐domain outcomes can further support site‐specific absolute risk calibration.

### 2.6 Modality Representation Imputation Enables Flexible Inference Under Missing Auxiliary Modalities

Reconstructed genomic and clinical‐report features exhibited clear linear correspondence with their original representations (Figure 5C). Genomic pathway features were recovered with high fidelity (cosine similarity 0.966, r=0.964, $R^{2}=0.854$), whereas clinical‐reports embeddings showed moderate agreement (cosine similarity 0.78, r=0.738, $R^{2}=0.422$). This asymmetry may partly reflect the more structured nature of pathway‐level genomic programs compared with the heterogeneous, partially observed nature of clinical narratives [19, 44]. These results indicate that the imputation modules function primarily as a regularizer, enforcing cross‐modal associations where they are biologically grounded while tolerating imperfect recovery where they are not [67]. Importantly, the module reconstructe latent token embeddings used by the frozen LLM backbone.

### 2.7 Prototype‐Guided Attention Reveals Interpretable Histopathologic Risk Patterns

The attention patterns of DAIMS were examined for pathological plausibility in representative cases from all three cohorts. Built on Virchow2 [68], the PGAF module learns prognostic prototypes (high‐risk and low‐risk) that aggregate WSI patches into slide‐level attention heatmaps, which can be overlaid on the original image.

#### 2.7.1 Attention Patterns in TCGA Source‐Domain Cases

In representative TCGA cases, DAIMS separated localized atypical foci from the broader tissue context rather than treating any isolated malignant patch as sufficient for a high‐risk call (Figure 6A,B). In the favorable‐outcome case (Figure 6A), high‐risk attention was mainly concentrated on well‐differentiated colorectal adenocarcinoma with crowded back‐to‐back glands, mild‐to‐moderate nuclear atypia, chronic inflammatory cells, and mild desmoplastic stromal reaction, whereas low‐risk activation preferentially covered benign colonic wall tissue with smooth muscle bundles and connective tissue. This pattern suggests that limited malignant morphology did not dominate the slide‐level risk estimate when embedded within a broader tissue landscape that remained largely non‐neoplastic [69, 70, 71].

**FIGURE 6:**
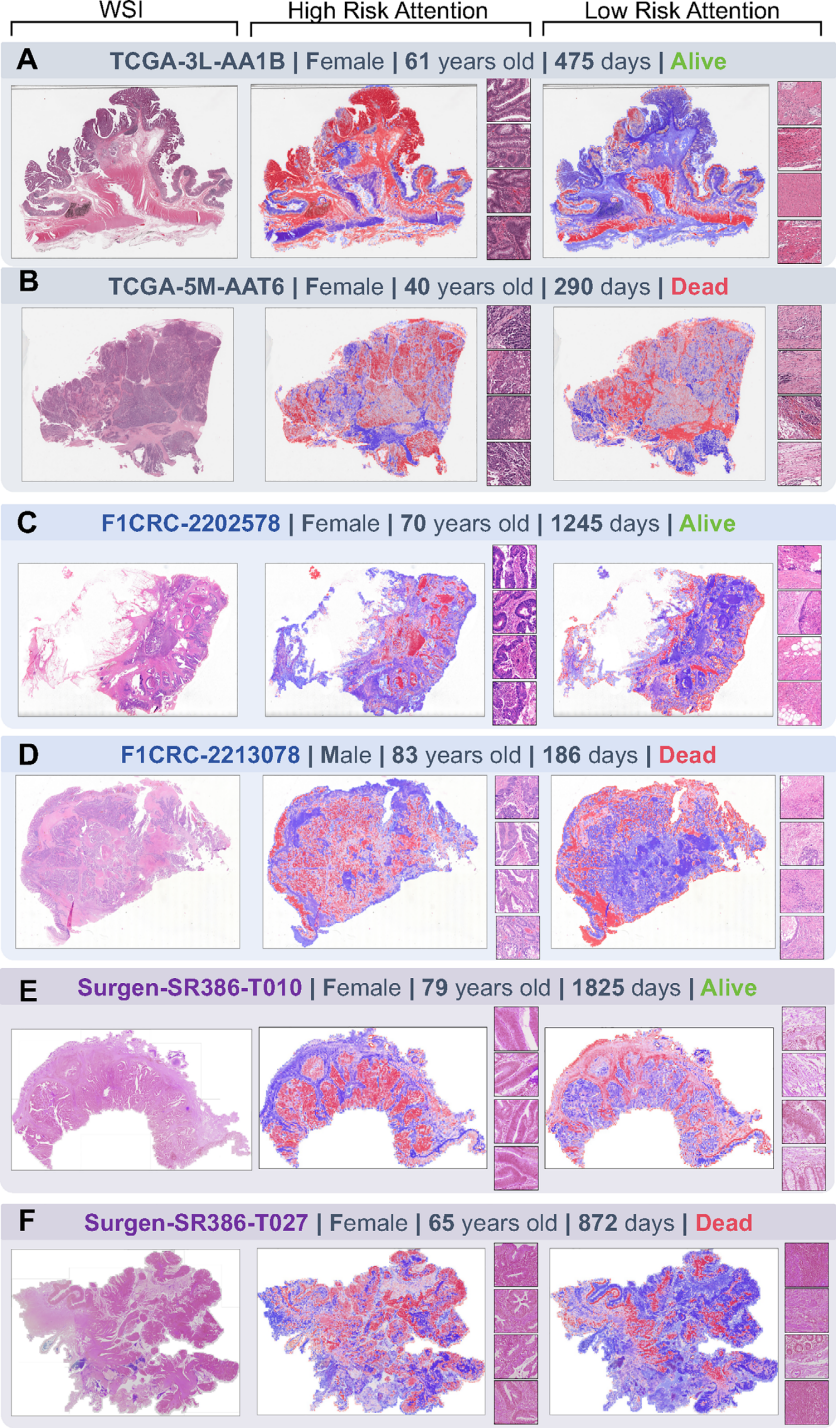
DAIMS attention visualization. Each panel shows the original WSI, high‐risk and low‐risk prototype attention heatmaps, and their highest‐attention regions. (A,B) are from the TCGA cohort: (A) is a surviving patient in whom high‐risk activation localizes to limited malignant glands while low‐risk activation covers broader benign or stromal tissue; (B) is a deceased patient with extensive high‐risk activation over poorly differentiated malignant regions and sparse low‐risk activation. (C,D) are from the TCGA‐trained model evaluated on F1CRC (surviving and deceased, respectively). (E,F) are from the TCGA‐trained model evaluated on SURGEN (also surviving and deceased). Across both external cohorts, DAIMS highlights morphologically coherent prognostic regions, demonstrating robust cross‐domain generalization.

In the poor‐outcome case (Figure 6B), the qualitative pattern reversed. High‐risk prototype activation became spatially extensive and concentrated on poorly differentiated adenocarcinoma with infiltrative sheets, nests, and cords of pleomorphic malignant cells, near‐complete loss of glandular architecture, and prominent desmoplastic stromal reaction, whereas low‐risk activation was relatively sparse. Thus, the key difference between the two samples is not only the presence of tumor, which can be found in both samples, but the predominance of high‐grade invasive morphology in the tissue landscape. This is consistent with the view that CRC outcomes are influenced by the global balance between malignant epithelial proliferation, stromal remodeling, and residual non‐neoplastic structures, rather than by any single isolated field of view [3, 10, 72].

#### 2.7.2 Attention Patterns Transfer Across Cohorts Despite Domain Shift

Cross‐domain heatmap analysis qualitatively supported the generalization results. When applied to representative cases from F1CRC and SURGEN, DAIMS preserved the same prototype logic observed on TCGA, with limited high‐risk activation concentrating on invasive adenocarcinoma regions and low‐risk activation preferentially covering fibroadipose stroma, reactive inflammatory background, necrosis, or only limited residual tumor components in regions with less aggressive morphology [10].

Across both external cohorts, surviving patient examples (Figure 6C,E) showed limited high‐risk prototype activation over gland‐forming adenocarcinoma regions, while low‐risk activation predominantly highlighted fibroadipose stroma, vessels, chronic inflammatory infiltrates, and other reactive non‐neoplastic tissue. In deceased patient examples (Figure 6D,F), DAIMS exhibited broader high‐risk activation across morphologically aggressive tumor regions. In F1CRC, these included cribriform or back‐to‐back malignant glands with necrosis, while in SURGEN they corresponded to more heterogeneous moderate‐to‐poorly differentiated adenocarcinoma. In contrast, low‐risk activation was largely restricted to benign background, extensive necrotic debris, or small residual lower‐grade tumor areas.

These examples show no exact color or texture matching across institutions due to differences in staining protocols and scanners, but consistent morphologic relationships. Across cases, DAIMS contrasts limited gland‐forming tumor with reactive stroma in lower‐risk samples vs. necrotic, pleomorphic, or disorganized carcinoma in higher‐risk samples [6, 53, 73]. The high‐ and low‐risk prototypes reflect differences in intratumoral heterogeneity, with higher‐risk cases showing greater spatial predominance of invasive tumor regions relative to benign architecture and gland‐forming components. Similar patterns are observed across three cohorts with distinct staining and demographic profiles. Expanded prototype‐level examples across predicted high‐ and low‐risk patients from F1CRC and SURGEN, with top‐attention patches shown for all four pathology foundation encoders, are provided in Figure S4.

### 2.8 Ablation Analysis Characterizes Modular Contributions

Four targeted ablation analyses were conducted to identify the components of DAIMS responsible for the observed prognostic performance, as well as their individual contribution to source‐domain discrimination and cross‐cohort generalization (Figure 7; Table S3).

**FIGURE 7:**
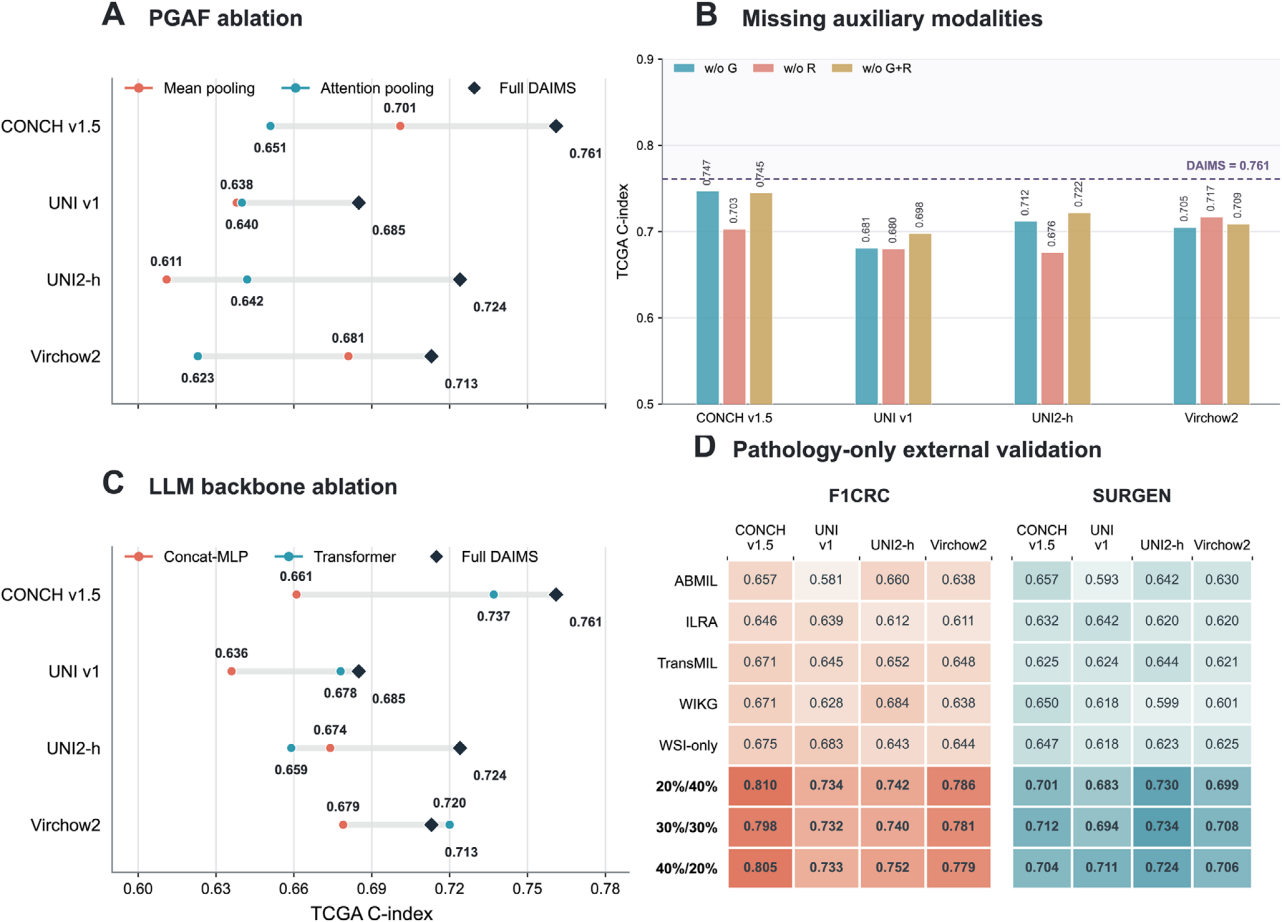
DAIMS ablation analysis. (A) PGAF was evaluated by replacing the prototype‐guided module with mean pooling or attention pooling, testing whether learned prototype‐guided token selection improves risk‐relevant aggregation. (B) Full DAIMS, trained with pathology, genomics, and clinical reports, reached the highest C‐index of 0.761 (dashed line) and exceeded every ablated configuration, which was trained without genomics, without clinical reports, or without both auxiliary modalities. All held‐out‐fold evaluations used pathology WSIs as the only direct input. This analysis therefore quantifies the contribution of auxiliary‐modality supervision to WSI‐only prognostic prediction. (C) The frozen LLM backbone was replaced with a concatenation‐MLP module or a transformer to evaluate the contribution of the sequence‐level fusion backbone. (D) The pathology‐only DAIMS branch was evaluated directly on external cohorts and compared with pathology‐only baselines and Full DAIMS adaptation settings.

#### 2.8.1 Effect of the Prototype‐Guided Attention Fusion Module

We replaced the PGAF module with mean pooling or attention pooling while keeping all other components unchanged. Both alternatives consistently underperformed the full DAIMS across all four encoders on TCGA (Figure 7A). With CONCH v1.5, the full PGAF module achieved a C‐index of 0.761, compared with 0.701 for mean pooling and 0.651 for attention pooling. Similar gaps were observed for UNI v1 (0.685 vs. 0.638 and 0.640), UNI2‐h (0.724 vs. 0.611 and 0.642), and Virchow2 (0.713 vs. 0.681 and 0.623). The attention‐pooling variant performed worse than mean pooling in most cases, suggesting that unstructured token aggregation loses prognostic information that the prototype‐guided mechanism preserves. These results indicate that PGAF contributes more than a generic compression step: the learned prototypes provide a structured space for selecting and organizing risk‐relevant tokens across modalities.

#### 2.8.2 Effect of Auxiliary Modalities

This analysis varied the modality combination during source‐domain training, while all held‐out‐fold evaluations used pathology WSIs as the only direct input. Across the four encoders, Full DAIMS (trained on complete modalities) achieved the highest C‐index of 0.761±0.114, compared with 0.711±0.024 for w/o G, 0.694±0.022 for w/o R, and 0.719±0.017 for w/o G+R (Figure 7B). Full DAIMS matched or exceeded the corresponding ablated configuration in all paired encoder‐by‐ablation comparisons. Overall, Full DAIMS provided the strongest and most consistent performance across encoders.

#### 2.8.3 Effect of the Frozen LLM Backbone

Across all four encoders, the frozen LLM backbone outperformed two simpler fusion alternatives (Figure 7C). Relative to the learnable transformer, the frozen backbone achieved higher C‐index values for CONCH v1.5, UNI v1, and UNI2‐h, whereas the learnable transformer was slightly higher for Virchow2 (0.720 vs. 0.713). These results support the frozen backbone as an effective parameter‐efficient fusion strategy in three of the four evaluated encoder configurations, rather than establishing universal superiority over trainable fusion. A concatenation‐MLP baseline achieved substantially lower C‐index values (0.661, 0.636, 0.674, 0.679), while a learnable transformer partially closed the gap (0.737, 0.678, 0.659, 0.720) and exceeded the frozen backbone on Virchow2 (0.720 vs. 0.713) but underperformed on the remaining three encoders. The consistent advantage of the frozen backbone indicates that pretrained token‐level interaction modeling generalizes effectively to heterogeneous multimodal sequences without additional training.

#### 2.8.4 Pathology‐Only Branch on External Cohorts

Finally, we evaluated the pathology‐only DAIMS branch on the two external cohorts to assess whether the multimodal‐trained backbone carries transferable risk information without target‐domain adaptation (Figure 7D). On F1CRC, the pathology‐only branch achieved C‐index values of 0.675, 0.683, 0.643, and 0.644 across the four encoders, ranking above most dedicated pathology‐only baselines under the same backbones. On SURGEN, it reached 0.647, 0.618, 0.623, and 0.625, remaining competitive with pathology‐only models. After lightweight adaptation, DAIMS further improved external performance across all encoders, reaching up to 0.810 on F1CRC and 0.734 on SURGEN. The gap between the full adapted DAIMS and the pathology‐only branch reflects the combined contribution of multimodal source‐domain supervision and target‐domain adaptation for cross‐cohort generalization. Interestingly, the pathology‐only branch, trained with multimodal supervision but deployed without adaptation, already outperforms pathology‐only models, indicating that multimodal training provides a lasting representational advantage.

## 3 Conclusions

In this study, we developed DAIMS, a domain‐adaptive incomplete multimodal stratification framework for colorectal cancer. By curating and analyzing whole‐slide images from three independent cohorts (TCGA, n=351; F1CRC, n=420; SURGEN, n=422), we assembled a heterogeneous multicenter dataset comprising more than 1,100 patients with variable acquisition conditions, outcome distributions, and molecular and clinical modality availability. DAIMS uses multimodal source‐domain supervision, PGAF, cross‐modal imputation, and lightweight WSI‐branch adaptation to address these real‐world shifts as a single transfer problem. Across evaluations spanning cohorts, treatment settings, disease stages, and molecular subtypes, the framework generally preserved prognostic stratification under heterogeneous deployment conditions. These findings suggest that histopathology can serve as a stable anchor modality, capturing clinically meaningful risk signals that remain informative despite cross‐cohort heterogeneity and incomplete multimodal inputs [49, 66, 73, 74].

Our results extend prior work showing that deep learning on histopathology images can support outcome‐aware patient stratification in colorectal cancer. However, many existing studies have been evaluated within single cohorts or under fully observed multimodal settings, which limits their utility in real‐world scenarios where target cohorts differ in acquisition pipeline, patient composition, and modality availability. DAIMS explicitly treats these factors as a domain adaptation problem. Incomplete multimodal learning is therefore not a parallel objective, but one mechanism that allows knowledge transfer from a fully paired source domain to WSI‐only external domains with minimal fine‐tuning and reduced dependence on cohort‐specific supervision. The consistent performance observed in both domain‐internal and domain‐external cohorts indicates that DAIMS learns shared prognostic representations rather than relying on dataset‐specific artifacts.

From a biological and pathological perspective, the robustness of DAIMS across treatment‐, stage‐, and genotype‐defined subgroups suggests that histopathology images encode latent prognostic information beyond conventional clinical labels. Morphological features reflecting tumor architecture, stromal composition, immune infiltration, and tumor–microenvironment interactions are known to influence disease progression and therapeutic response in colorectal cancer. DAIMS appears to capture such signals that remain informative within stage‐ and treatment‐defined strata, as evidenced by its ability to stratify patients within standardized chemotherapy regimens and genetically defined subgroups, including BRAF‐mutant, KRAS‐mutant, and MMR‐deficient tumors [75]. These observations support the view that histopathology contains rich prognostic information that not only complements molecular profiling but may also partially compensate for it when molecular assays are unavailable.

Across all tested encoder architectures, including CONCH v1.5 [8], UNI v1, UNI2‐h [39], and Virchow2 [68], DAIMS outperformed pathology‐only models in most evaluated settings, including ABMIL [76], ILRA [77], TransMIL [78], and WIKG [79], as well as previously reported multimodal methods, including CMTA [80], MOTCAT [31], and SurvPath [81]. This advantage was preserved under modality‐availability shifts, where DAIMS maintained robust discrimination and yielded absolute C‐index gains of 0.04–0.05 over the strongest baselines in TCGA internal evaluation and mean gains of 10.4% and 6.6% on F1CRC and SURGEN, respectively. The frozen LLM backbone served as a fixed sequence‐level fusion to organize heterogeneous pathology, genomic, and clinical tokens without adding extra trainable parameters, and PGAF improved risk‐relevant token aggregation. The validity of this interpretation is corroborated by the performance drop when replacing the frozen backbone with a concatenation‐MLP or a learnable transformer. For target‐domain adaptation, DAIMS restricts pseudo‐label‐based updates to the WSI adapter and prediction head, while fixing the pathology encoder, imputation modules, and LLM backbone. This limited update space reduces the risk of pseudo‐label drift. The improved post‐adaptation risk‐distribution overlap shows that adaptation can recalibrate relative‐risk organization rather than amplifying unstable pseudo‐label noise.

The clinical implications of these findings are substantial. DAIMS offers a practical approach for risk stratification using routinely acquired pathology slides, enabling timely prognostic assessment even in resource‐limited settings or when molecular profiling is unavailable or delayed. By identifying overall prognostic risk within stage‐ and treatment‐defined groups, DAIMS has supported risk stratification across real‐world care settings that may include concurrent procedures and treatments, such as surgery, chemotherapy, radiation therapy, and other pharmacological treatments. Future studies with standardized treatment‐response endpoints will extend this framework toward treatment‐benefit assessment. Importantly, DAIMS is not intended to replace molecular testing, but rather to complement existing diagnostic pipelines by providing an additional layer of risk information. DAIMS has provided cohort‐relative prognostic rankings, consistent with recent pathology‐based prognostic studies that have used model‐derived scores for risk stratification across cohorts [20, 21, 33, 82]. Site‐specific calibration with target‐domain outcomes can further support absolute risk estimation and clinical deployment.

Several limitations should be acknowledged. The study cohorts differed in demographic composition: TCGA was predominantly White, F1CRC was entirely East Asian, and SURGEN was predominantly Caucasian. In addition, the representation of some molecular subgroups, including *BRAF*‐mutant and dMMR patients, was limited. Future validation in larger and more demographically diverse cohorts with broader representation of molecular subtypes will help further characterize the generalizability of DAIMS across these patient populations. External validation also involves endpoint heterogeneity, as TCGA‐based survival definitions may not fully align with those in external cohorts, introducing both covariate and label‐space shifts. In addition, the WSI‐only deployment setting creates an information bottleneck, as pathology cannot fully substitute for molecular or clinical variables in all cases. The contribution of modality ablation analysis showed that complete multimodal supervision provided the strongest overall performance across pathology encoders for WSI‐only inference. Future work will investigate adaptive cross‐modal alignment and modality weighting across encoder architectures. Finally, the observational design precludes causal interpretation of the learned features. Future work will focus on harmonizing endpoints across larger multi‐center datasets, incorporating emerging modalities such as spatial transcriptomics [83], improving interpretability, and extending DAIMS to other cancer types.

In summary, DAIMS provides an explainable, domain‐adaptive, and modality‐agnostic framework for colorectal cancer patient stratification, capable of delivering robust prognostic assessment under heterogeneous real‐world conditions. These findings underscore the potential of histopathology‐centered multimodal learning, organized around domain adaptation, to advance precision oncology and enable potentially useful prognostic risk assessment, subject to prospective validation and site‐specific calibration in real‐world practice.

## 4 Experimental Methods

### 4.1 Overview of DAIMS Framework

We introduce DAIMS, a lightweight domain‐adaptive framework for real‐world CRC patient stratification under cross‐cohort modality and modality‐availability shift. DAIMS is trained on the fully paired TCGA‐CRC dataset, which includes WSIs, structured clinical reports, and genomic pathway features. WSIs were processed using TRIDENT, a scalable WSI‐processing toolkit, to perform tissue segmentation, patch tiling, and patch‐level feature extraction with pathology foundation models including UNI v1, UNI2‐h, Virchow2, and CONCH v1.5 [84, 85]. Encoder‐specific preprocessing, native feature dimensions, and downstream harmonization are summarized in Table S4. Clinical reports are embedded using BioBERT [86] after LLM‐based structuring (See Table S16), and pathway activity profiles are transformed via self‐normalizing networks [87]. DAIMS employs three Prototype‐Guided Aggregation and Fusion (PGAF) modules to select discriminative tokens from each modality and map them into a shared latent representation. To handle incomplete modalities as a deployment‐domain shift, Prototype‐Guided Cross‐Modal Representation Imputation modules learn to reconstruct clinical and genomic token embeddings directly from WSI features, enabling consistent multimodal supervision even in pathology‐only cohorts. When evaluated on the external F1CRC and SURGEN cohorts, DAIMS performs efficient domain adaptation by updating only a small subset of parameters, including the WSI adapter and the final prediction head. Iterative pseudo‐labeling further aligns latent representations to cohort‐specific staining and scanner characteristics.

### 4.2 Prototype‐Based Feature Selection

To distill informative morphological patterns from multimodal embeddings, we employ a prototype‐based feature selection module, consisting of (i) a learnable Gaussian prototype memory bank and (ii) a differentiable feature aggregation mechanism. Given input tokens $x\in R^{B\times L\times d}$, a projection layer encodes them into latent representations $z\in R^{B\times L\times D}$.

Unlike conventional class‐specific prototypes, we define Gaussian prototypes corresponding to distinct risk stratification, each parameterized by $\left(\mu_{k},\sigma_{k}\right)$ to capture the distribution of morphological patterns associated with a predicted risk level. During training, proxy samples are drawn as

$${\tilde{z}}_{k}=\mu_{k}+\sigma_{k}⊙\varepsilon ,\;\varepsilon ∼N\left(0,I\right),$$

to provide distributional regularization in the contrastive loss. During inference, the deterministic means $\mu_{k}$ are used directly, ensuring stable and reproducible aggregation.

To compress the L raw tokens into a compact set of K semantically structured representations, we introduce K learnable aggregation centroids $C=\{c_{1},\text{…},c_{K}\}$, which serve as trainable slots for the downstream LLM. Token‐centroid similarity determines the attention weights:

$$\alpha_{i,j,k}=\frac{\exp(\left\langle z_{i,j},c_{k}\right\rangle /\tau_{\text{att}})}{\sum_{j^{\prime }=1}^{L}\exp\left(\left\langle z_{i,j^{\prime }},c_{k}\right\rangle /\tau_{\text{att}}\right)},$$

and the aggregated features are obtained as

$$z_{i,k}^{\text{agg}}=\sum_{j=1}^{L}\alpha_{i,j,k}\;z_{i,j},\;z^{\text{agg}}\in R^{B\times K\times D}.$$

The global sequence‐level representation is then defined as the mean of the aggregated slots ${\bar{z}}^{\text{agg}}$. The aggregated representation is aligned with its corresponding risk‐stratified prototype through a contrastive objective:

$$L_{\text{proto}}=-\log\frac{\exp(\left\langle {\bar{z}}^{\text{agg}},\mu_{p(i)}\right\rangle /\tau_{\text{proto}})}{\sum_{k}\exp\left(\left\langle {\bar{z}}^{\text{agg}},\mu_{k}\right\rangle /\tau_{\text{proto}}\right)},$$

where p(i) maps each sample to the prototype of its predicted risk label derived from clinical event y and survival status c. This loss encourages clustering around the appropriate prototype, improving discriminability and stability under domain shift.

### 4.3 Modality Representation Imputation

In real‐world clinical datasets, genomic and clinical modalities are often unavailable in the target domain, while WSIs are nearly always available. We therefore treat modality incompleteness as a modality‐availability shift within the domain adaptation setting. To enable pathology‐only inference while still leveraging multimodal supervision during training, we introduce the Prototype‐Guided Cross‐Modal Representation Imputation module. The core idea is to use the stable target‐modality prototypes learned in the previous stage as semantic anchors to guide the generation of surrogate latent tokens for unavailable modalities, rather than explicitly reconstructing the original signals.

Given a source modality feature sequence $s\in R^{B\times S\times d_{s}}$ (e.g., WSI features), DAIMS first projects it into the target latent space via a linear mapping $z_{s}=W_{s}s$. A two‐stage cross‐attention stack then transfers these source features into the structural space of the target modality. The first attention layer aligns $z_{s}$ with the frozen target prototypes ${\left\{\mu_{k}\right\}}_{k=1}^{P}$:

$$h_{1}=\mathrm{LN}\left(z_{s}+\gamma_{1}\mathrm{MHA}\left(\text{query}=z_{s},\text{key}=\mu ,\text{value}=\mu \right)\right),$$

where $\gamma_{1}$ is a learnable residual scaling parameter. Subsequently, K learnable query slots $q\in R^{K\times d_{t}}$ query the aligned representation $h_{1}$ to construct target‐shaped latent tokens:

$$h_{2}=\mathrm{LN}\left(q+\gamma_{2}\mathrm{MHA}\left(\text{query}=q,\text{key}=h_{1},\text{value}=h_{1}\right)\right).$$

A decoder then refines $h_{2}$ to produce the reconstructed target tokens $\hat{t}\in R^{B\times K\times d_{t}}$. To ensure the fidelity of the imputation when ground‐truth target tokens $t_{gt}$ are available, we employ a supervised loss based on Hungarian matching. Specifically, we solve a linear sum assignment problem to find the optimal permutation π that minimizes the bipartite matching cost:

$$L_{sup}=\min_{\pi \in \Pi_{K}}\frac{1}{K}\sum_{i=1}^{K}{∥{\hat{t}}_{i}-t_{gt,\pi (i)}∥}^{2},$$

which accounts for potential semantic permutations among the learnable slots.

Finally, DAIMS combines the reconstructed tokens with a source‐derived baseline using an adaptive gating mechanism computed from the variability of the source features. This enables the model to generate high‐quality surrogate latent representations for missing modalities during inference using only WSIs, without requiring explicit recovery of the original modality signals.

### 4.4 Cross‐Cohort Validation and Domain Adaptation

For external validation, we benchmarked DAIMS against pathology‐only baselines (ABMIL, TransMIL, ILRA, and WIKG) on independent cohorts with differences in staining, scanning resolution, patient composition, survival distributions, and modality availability. We initialized adaptation with TCGA‐trained checkpoints and adapted each external cohort independently using unlabeled target WSIs. DAIMS produced continuous cohort‐relative risk scores. We evaluated within‐cohort discrimination using the C‐index and defined high‐ and low‐risk groups using the median predicted risk score within each cohort. Target‐domain event times and censoring indicators were not used during adaptation or score fusion.

At each adaptation iteration, the pre‐update survival model generated a pseudo‐event bin $\tilde{y}$ and a continuous relative risk score for each target WSI. A bootstrap WSI view assessed prediction consistency and guided target‐case selection. In parallel, a frozen censor predictor trained on TCGA generated a soft censoring probability $\hat{p}\left(c=1\right)$ for each target case.

We used eight adaptation iterations with four epochs per iteration, a learning rate of $2\times 10^{-5}$, a WSI batch size of one, and up to 4,096 patch embeddings per slide. We updated the prediction head and WSI adapter, while keeping the pathology encoder, modality‐imputation modules, and frozen LLM backbone fixed. At each iteration, we selected up to 50% of target cases with consistent risk predictions and optimized the following soft‐censoring survival loss:

(1) $$L_{\mathrm{surv}}\;=\;\left(1-\hat{p}\left(c=1\right)\right)\;L_{\mathrm{NLL}}\left(\tilde{y},\;c=0\right)\;+\;\hat{p}\left(c=1\right)\;L_{\mathrm{NLL}}\left(\tilde{y},\;c=1\right).$$

The final target‐domain score was obtained through equal‐weight cohort‐rank fusion. We ranked the adapted survival risk and frozen soft‐censoring probability within each target cohort and averaged the two ranks.

### 4.5 Model Visualization and Interpretation

We further validated that DAIMS captures biologically meaningful information in colorectal cancer by developing an interpretation pipeline based on the PGAF module. Specifically, cross‐attention weights aligning raw pathology tokens with risk‐stratified prototypes were used to identify the most informative morphological patterns. By mapping attention scores from the highest‐ and lowest‐risk prototypes back to the original WSI coordinates, we generated high‐resolution heatmaps that highlight tumor regions, stromal structures, and immune cell arrangements associated with poor or favorable prognosis. These visualizations, grounded in the Virchow2 foundation model, allow us to verify that DAIMS encodes transferable morphological signals rather than institution‐specific appearance.

### 4.6 Computational Trade‐Off

To estimate practical deployment cost, we summarized model size, trainable parameters, GPU memory usage, and inference latency under a unified evaluation setting (see Table 2). Each modality is passed through a lightweight adapter and fused using a frozen BioBERT‐Base v1.2 backbone [86]. This encoder‐only Transformer comprises 12 layers, 12 attention heads, a hidden dimension of 768, and approximately 108.3 million parameters in our implementation. All backbone parameters were frozen during source‐domain training and target‐domain adaptation, and the model was used as a fixed sequence‐level fusion module rather than as a generative language model. Across the evaluated encoders, the total parameter count remained approximately 171M, while only about 63M parameters were trainable because the language backbone was frozen. Memory use was about 0.64 GB. Inference latency was measured with a batch size of one on NVIDIA L20 (48 GB), NVIDIA RTX 6000 (96 GB), and NVIDIA H200 GPUs, using up to 4,096 pre‐extracted WSI patch embeddings per slide. The reported latency reflects patient‐level DAIMS inference after patch features are available and excludes tissue segmentation, patch tiling, and pathology‐foundation‐model feature extraction. Across the evaluated model configurations, mean per‐slide latency ranged from approximately 30 to 34 ms. For the same configuration, mean latency differed by less than 1 ms across GPU platforms.

**TABLE 2:** Computational trade‐off of DAIMS across WSI encoders under identical settings.

Patch Encoder	Params (M)	Trainable (M)	Memory (GB)	Inference (ms)	Throughput ($s^{-1}$)
UNI v1	171.23	62.89	0.64	32.35 ± 9.00	30.91
UNI2‐h	171.49	63.15	0.64	32.28 ± 9.20	30.98
CONCH v1.5	171.10	62.76	0.64	29.88 ± 10.06	33.47
Virchow2	172.02	63.67	0.64	33.58 ± 8.82	29.78
**Average**	171.46	63.12	0.64	32.02	31.29

## Author Contributions

J. Z, Z.J., and C. C. conceived the study, designed the methodology, performed experiments, analyzed the data, and drafted the original manuscript. Z.J., J.Z., C.C., C. H., Y.W. contributed to the original draft. All authors contributed to results interpretation, illustration, critically revised the manuscript, and approved the final version for publication. J.Z. oversaw the entire project. C.C., Y. J., J.Z. and C.D. provided clinical interpretation.

## Funding

J.Z. is supported by Cancer Research Foundation, Nvidia Academic Grant Award, Anthropic AI for Science Program, and the University of Chicago Provost Award.

## Ethics Statement

This study analyzed de‐identified human data from three colorectal cancer cohorts. The TCGA and SURGEN datasets were obtained from publicly available repositories and used in accordance with their respective data access policies. The F1CRC cohort was provided by The First Affiliated Hospital of University of South China as a retrospective, de‐identified dataset. Ethical approval for the use of the F1CRC cohort was granted by the Institutional Review Board of The First Affiliated Hospital of University of South China (approval number: 2025LL1009001). Given the retrospective nature of the study and the use of de‐identified data, the requirement for informed consent was waived by the Institutional Review Board. All research was conducted in accordance with the principles of the Declaration of Helsinki.

## Consent

Not applicable because the manuscript does not contain identifiable individual data.

## Conflicts of Interest

J. Zhao reports an advisory role with and receipt of consulting fees and equity from Encapsulate Inc.; equity in Alvus Health Inc.; and service on the research advisory board of Shriners Children's. S. Yang and L. Lu are employed by Ant Group and reports shareholdings.

## Supporting information


**Supporting File**: dvs78019‐sup‐0001‐SuppMat.zip.

## Data Availability

This study utilized whole‐slide images (WSIs) and associated clinical data from three independent cohorts. Colorectal cancer (CRC) data from The Cancer Genome Atlas (TCGA) were obtained through the Genomic Data Commons (GDC) Data Portal (https://portal.gdc.cancer.gov/). The SURGEN dataset is publicly available from the EMBL–EBI BioStudies repository under accession number S‐BIAD1285 and can be accessed at https://www.ebi.ac.uk/biostudies/bioimages/studies/S‐BIAD1285. The data (F1CRC) that support the findings of this study are available on request from the corresponding author. The data are not publicly available due to privacy or ethical restrictions.
