# Supplementary material for: A Generalizable Multimodal Model for Treatment‐Stratified Risk and Survival Assessment under Real‐World Constraints: A Multi‐Center Study of Colorectal Cancer
Source: Adv Sci (Weinh). 2026 Sep 29:e78019. Online ahead of print. doi: 10.1002/advs.78019 (PMC13624312; doi:10.1002/advs.78019)

Sensitivity of DAIMS to WSI Patch Budget at Inference

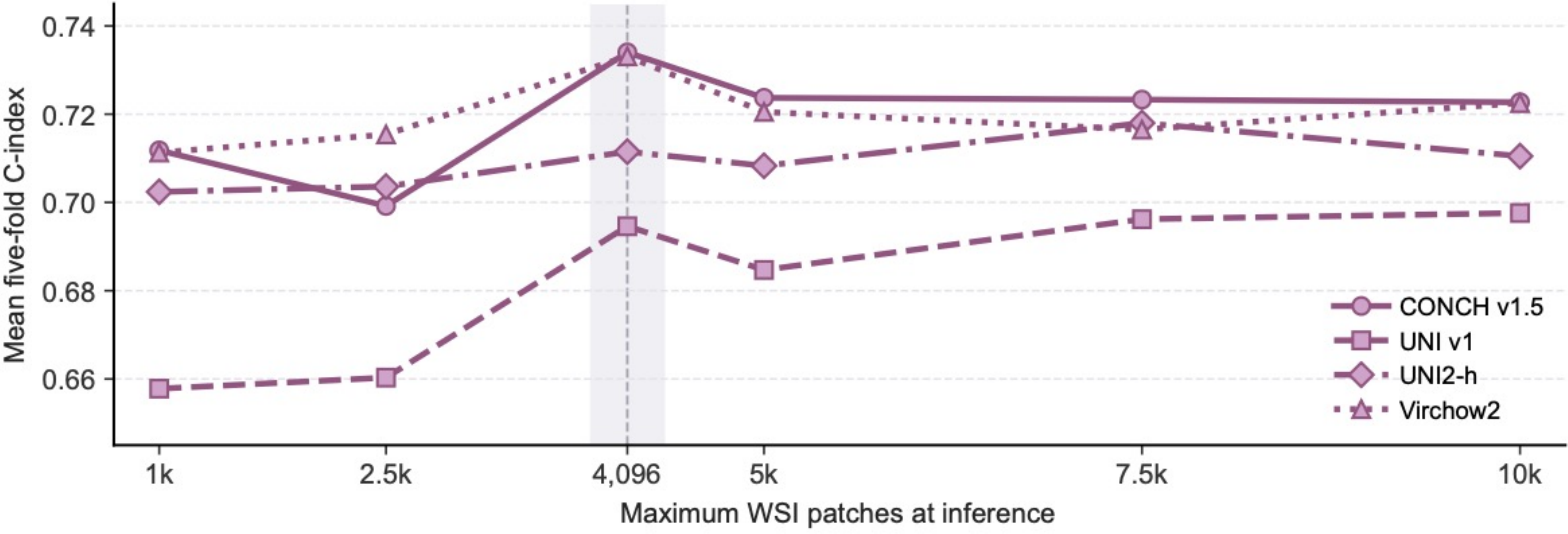

Supplement: Supplementary file 1 [file ADVS-9999-e78019-s001.zip › figs/s5.pdf]
