# Supplementary material for: A Generalizable Multimodal Model for Treatment‐Stratified Risk and Survival Assessment under Real‐World Constraints: A Multi‐Center Study of Colorectal Cancer
Source: Adv Sci (Weinh). 2026 Sep 29:e78019. Online ahead of print. doi: 10.1002/advs.78019 (PMC13624312; doi:10.1002/advs.78019)
Supplement: Supplementary file 1 [file ADVS-9999-e78019-s001.zip › figs/s4.pdf]

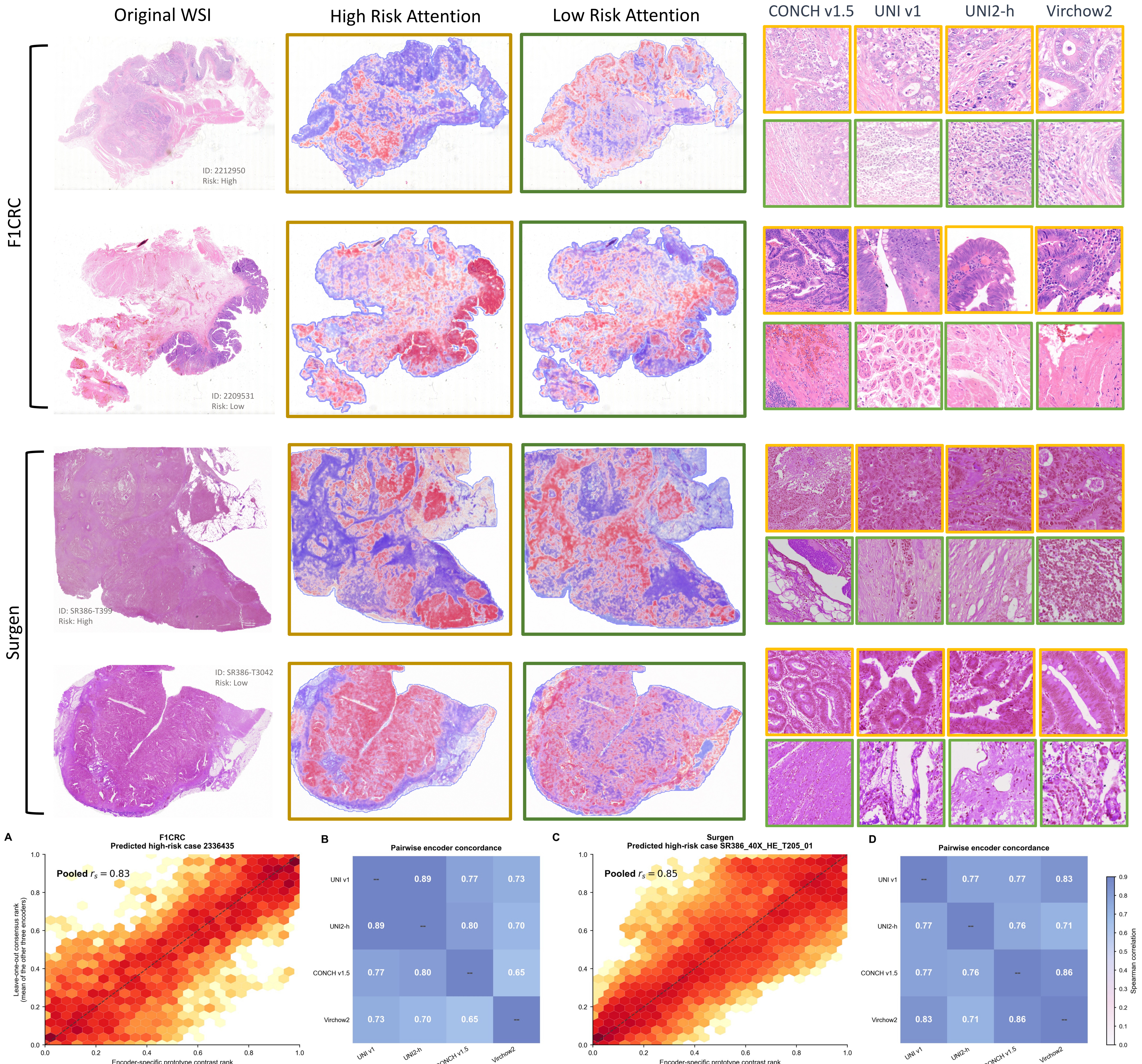
Original WSI
High Risk Attention
Low Risk Attention
CONCH v1.5
UNI v1
UNI2-h
Virchow2
F1CRC
ID: 2212950
Risk: High
ID: 2209531
Risk: Low
Surgen
ID: SR386-T399
Risk: High
ID: SR386-T3042
Risk: Low
A
F1CRC
Predicted high-risk case 2336435
Pooled $r_s = 0.83$
Leave-one-out consensus rank
(mean of the other three encoders)
Encoder-specific prototype contrast rank
B
Pairwise encoder concordance
UNI v1
UNI2-h
CONCH v1.5
Virchow2
-- 0.89 0.77 0.73
0.89 -- 0.80 0.70
0.77 0.80 -- 0.65
0.73 0.70 0.65 --
C
Surgen
Predicted high-risk case SR386_40X_HE_T205_01
Pooled $r_s = 0.85$
Encoder-specific prototype contrast rank
D
Pairwise encoder concordance
-- 0.77 0.77 0.83
0.77 -- 0.76 0.71
0.77 0.76 -- 0.86
0.83 0.71 0.86 --
Spearman correlation
