# Supplementary material for: A Generalizable Multimodal Model for Treatment‐Stratified Risk and Survival Assessment under Real‐World Constraints: A Multi‐Center Study of Colorectal Cancer
Source: Adv Sci (Weinh). 2026 Sep 29:e78019. Online ahead of print. doi: 10.1002/advs.78019 (PMC13624312; doi:10.1002/advs.78019)
Supplement: Supplementary file 1 [file ADVS-9999-e78019-s001.zip › figs/s3.pdf]

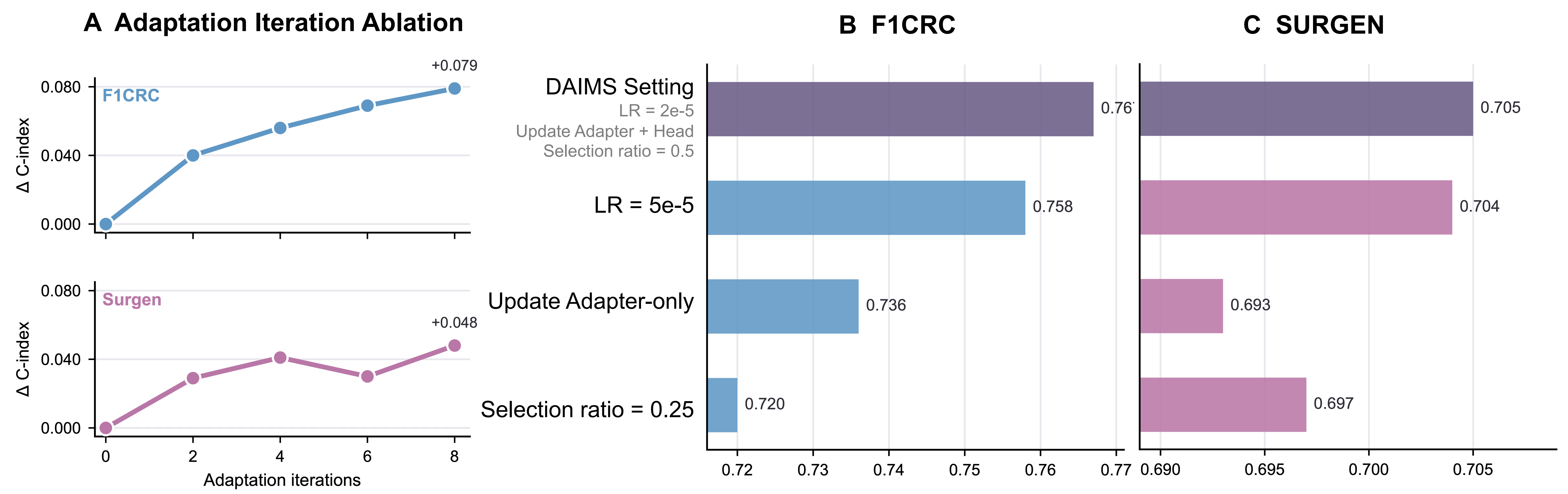
A Adaptation Iteration Ablation
F1CRC
+0.079
0.080
0.040
0.000
Δ C-index
Surgen
+0.048
0.080
0.040
0.000
Δ C-index
0
2
4
6
8
Adaptation iterations
B F1CRC
C SURGEN
DAIMS Setting
LR = 2e-5
Update Adapter + Head
Selection ratio = 0.5
LR = 5e-5
Update Adapter-only
Selection ratio = 0.25
0.76
0.758
0.736
0.720
0.72
0.73
0.74
0.75
0.76
0.77
0.705
0.704
0.693
0.697
0.690
0.695
0.700
0.705
