# Supplementary material for: A Generalizable Multimodal Model for Treatment‐Stratified Risk and Survival Assessment under Real‐World Constraints: A Multi‐Center Study of Colorectal Cancer
Source: Adv Sci (Weinh). 2026 Sep 29:e78019. Online ahead of print. doi: 10.1002/advs.78019 (PMC13624312; doi:10.1002/advs.78019)
Supplement: Supplementary file 1 [file ADVS-9999-e78019-s001.zip › figs/s2.pdf]

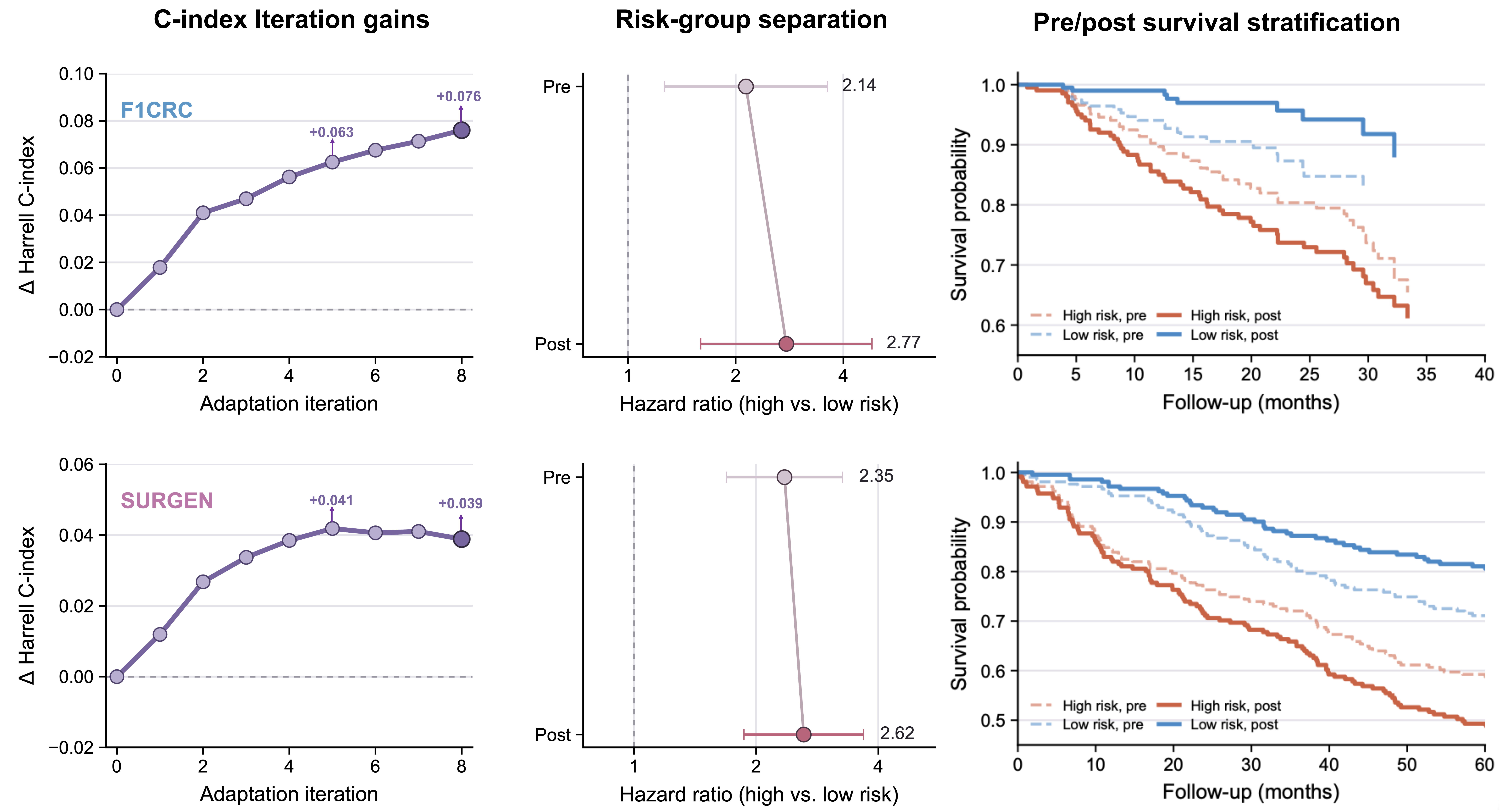

C-index Iteration gains
Risk-group separation
Pre/post survival stratification
F1CRC
+0.063
+0.076
Δ Harrell C-index
Adaptation iteration
Pre
Post
2.14
2.77
Hazard ratio (high vs. low risk)
Survival probability
Follow-up (months)
High risk, pre
High risk, post
Low risk, pre
Low risk, post
SURGEN
+0.041
+0.039
2.35
2.62
