# Supplementary material for: A Generalizable Multimodal Model for Treatment‐Stratified Risk and Survival Assessment under Real‐World Constraints: A Multi‐Center Study of Colorectal Cancer
Source: Adv Sci (Weinh). 2026 Sep 29:e78019. Online ahead of print. doi: 10.1002/advs.78019 (PMC13624312; doi:10.1002/advs.78019)
Supplement: Supplementary file 1 [file ADVS-9999-e78019-s001.zip › figs/s1.pdf]

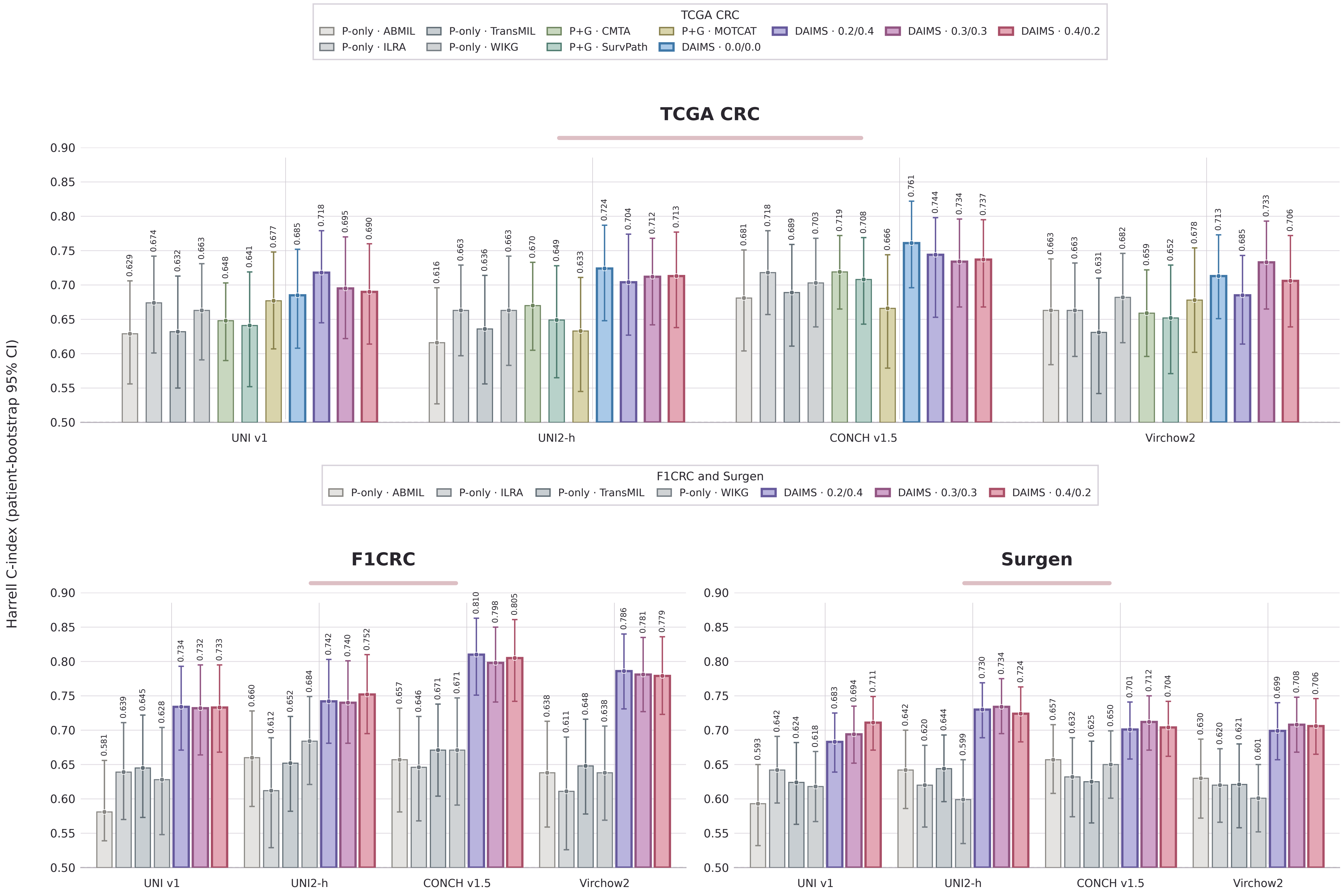

Bars begin at C-index 0.50; whiskers denote patient-level bootstrap 95% confidence intervals.
